# *In-silico* evaluation of adenoviral COVID-19 vaccination protocols: Assessment of immunological memory up to 6 months after the third dose

**DOI:** 10.3389/fimmu.2022.998262

**Published:** 2022-10-24

**Authors:** Paola Stolfi, Filippo Castiglione, Enrico Mastrostefano, Immacolata Di Biase, Sebastiano Di Biase, Gianna Palmieri, Antonella Prisco

**Affiliations:** ^1^ Institute for Applied Computing, National Research Council of Italy, Rome, Italy; ^2^ MeriGen Res, Naples, Italy; ^3^ Institute of Biosciences and BioResources, National Research Council, Naples, Italy; ^4^ Institute of Genetics and Biophysics, National Research Council, Naples, Italy

**Keywords:** immunological memory, adenoviral COVID-19 vaccine, booster, *in silico*, agent-based modeling (ABM), simulation, anti-vector immunity

## Abstract

**Background:**

The immune response to adenoviral COVID-19 vaccines is affected by the interval between doses. The optimal interval is unknown.

**Aim:**

We aim to explore in-silico the effect of the interval between vaccine administrations on immunogenicity and to analyze the contribution of pre-existing levels of antibodies, plasma cells, and memory B and T lymphocytes.

**Methods:**

We used a stochastic agent-based immune simulation platform to simulate two-dose and three-dose vaccination protocols with an adenoviral vaccine. We identified the model’s parameters fitting anti-Spike antibody levels from individuals immunized with the COVID-19 vaccine AstraZeneca (ChAdOx1-S, Vaxzevria). We used several statistical methods, such as principal component analysis and binary classification, to analyze the correlation between pre-existing levels of antibodies, plasma cells, and memory B and T cells to the magnitude of the antibody response following a booster dose.

**Results and conclusions:**

We find that the magnitude of the antibody response to a booster depends on the number of pre-existing memory B cells, which, in turn, is highly correlated to the number of T helper cells and plasma cells, and the antibody titers. Pre-existing memory T cytotoxic cells and antibodies directly influence antigen availability hence limiting the magnitude of the immune response. The optimal immunogenicity of the third dose is achieved over a large time window, spanning from 6 to 16 months after the second dose. Interestingly, after any vaccine dose, individuals can be classified into two groups, *sustainers* and *decayers*, that differ in the kinetics of decline of their antibody titers due to differences in long-lived plasma cells. This suggests that the *decayers* may benefit from a tailored boosting schedule with a shorter interval to avoid the temporary loss of serological immunity.

## 1 Introduction

Most COVID-19 vaccines are given in a two-dose primary schedule, whereas additional booster doses may be required to maintain immunity. The time interval between vaccine administrations can greatly affect the logistics of the vaccination campaign and its efficacy (1–3). The effect of the dosing interval on COVID-19 vaccine efficacy has not been specifically tested in Randomized Clinical Trials (RCT), however, available data suggest that longer intervals between the first and second dose result in higher antibody titers (4). Since the investigation of vaccine dosing protocol in RCT is limited by feasibility issues, in-silico modeling can make an important contribution to the field, allowing the extensive exploration of different schedules and the identification of the immunological variables that correlate with the endpoints of interest (5, 6). In this study, we utilized stochastic agent-based modeling to study the effect of the dosing protocol on the immune response to an adenoviral vaccine. Agent-based models exhibit emergent properties and thus can also lead to the discovery of patterns in the complex behavior of the immune system.

Adenoviral vaccines are less expensive and easier to store and transport than mRNA vaccines. On the other hand, vectored vaccines are not expected to be ideal for repeated administration (7). Their efficacy can be reduced by at least two factors: i) a preexistent antibody response to the vector that interferes with transduction (anti-vector immunity) (8) and/or ii) a preexistent cytotoxic T cell response against either the vector or the insert that limits the persistence of transduced cells (9). The determination of the optimal time interval between doses of adenoviral vaccines is still an unresolved question, and not enough is known about tailored schedules for groups that might need additional doses, such as the elderly or immunocompromised individuals.

The COVID-19 vaccine AstraZeneca is based on a chimpanzee adenovirus, utilized to generate the vector ChAdOx1. A low prevalence of anti-vector neutralizing antibodies has been observed in humans (10). Nevertheless, among the participants of clinical trials, before vaccination, some had high titer (IC50 > 200) or low titer (IC50< 200) neutralizing antibodies against ChAdOx1 (11, 12). The first dose induced anti-vector neutralizing antibodies that persisted until the last assessed time point (84 days) but did not prevent boosting (12). Indeed, clinical trials have shown that repeated use of AstraZeneca is effective: the second dose induces a marked surge of antibody titers and increased protection (12–14). The vaccine has been approved as a two-dose vaccine, with an inter-dose interval of 4 to 12 weeks (15). A clinical study analyzed immune responses to the AstraZeneca vaccine over an extended interval between the first and second administration, and after a third dose. It was shown that a longer inter-dose interval leads to higher antibody titers and that a third dose greatly increases antibody titers (11). Interestingly, in aged individuals, one dose of either the mRNA-based Pfizer vaccine or the adenoviral-vectored AstraZeneca vaccine elicits similar antibody levels on day 35 (16), whereas the second homologous dose, given after an 8–12 week interval, results in higher antibody titers in those vaccinated with the mRNA vaccine (17). This observation is consistent with the idea that the immunogenicity of the first dose of Pfizer and AstraZeneca are comparable, whereas the immunogenicity of the second dose of the adenoviral vaccine is reduced, with the caveat that the different kinetics of the antibody responses to the first dose may limit the significance of the day 35 comparison (17). Individuals vaccinated with a first dose of AstraZenca show a robust immune response when the second dose is an mRNA vaccine (18). A comparison of homologous and heterologous dosing protocols showed that after one dose of AstraZeneca, a second heterologous dose elicits higher antibody titers (19–21). After two doses of AstraZeneca, a third homologous dose elicits lower anti-Spike antibody titers than a third heterologous dose of mRNA vaccine (22).

By comparing different vaccination protocols we aim to investigate the effect of the interval between adenoviral vaccine doses by means of a stochastic agent-based immune simulation platform. We adjusted the model parameters using anti-Spike antibody data from individuals immunized with the COVID-19 vaccine AstraZeneca, from two sets of data, namely i) the “Vaxab dataset”, a retrospective observational study on anti-RBD-Spike total antibodies in individuals vaccinated against COVID-19 in Naples, Italy, and ii) published serological data from the COV001 and COV002 trials (11).

In the following sections, we describe the clinical data used to identify the parameters of the computational model, the model itself, the statistical procedures used to set the parameters, the definition of the numerical experiments to be conducted in-silico, and, finally, the analysis of the results obtained and the conclusions drawn.

## 2 Material and methods

### 2.1 Dataset 1: The observational study Vaxab

**Figure 1:**
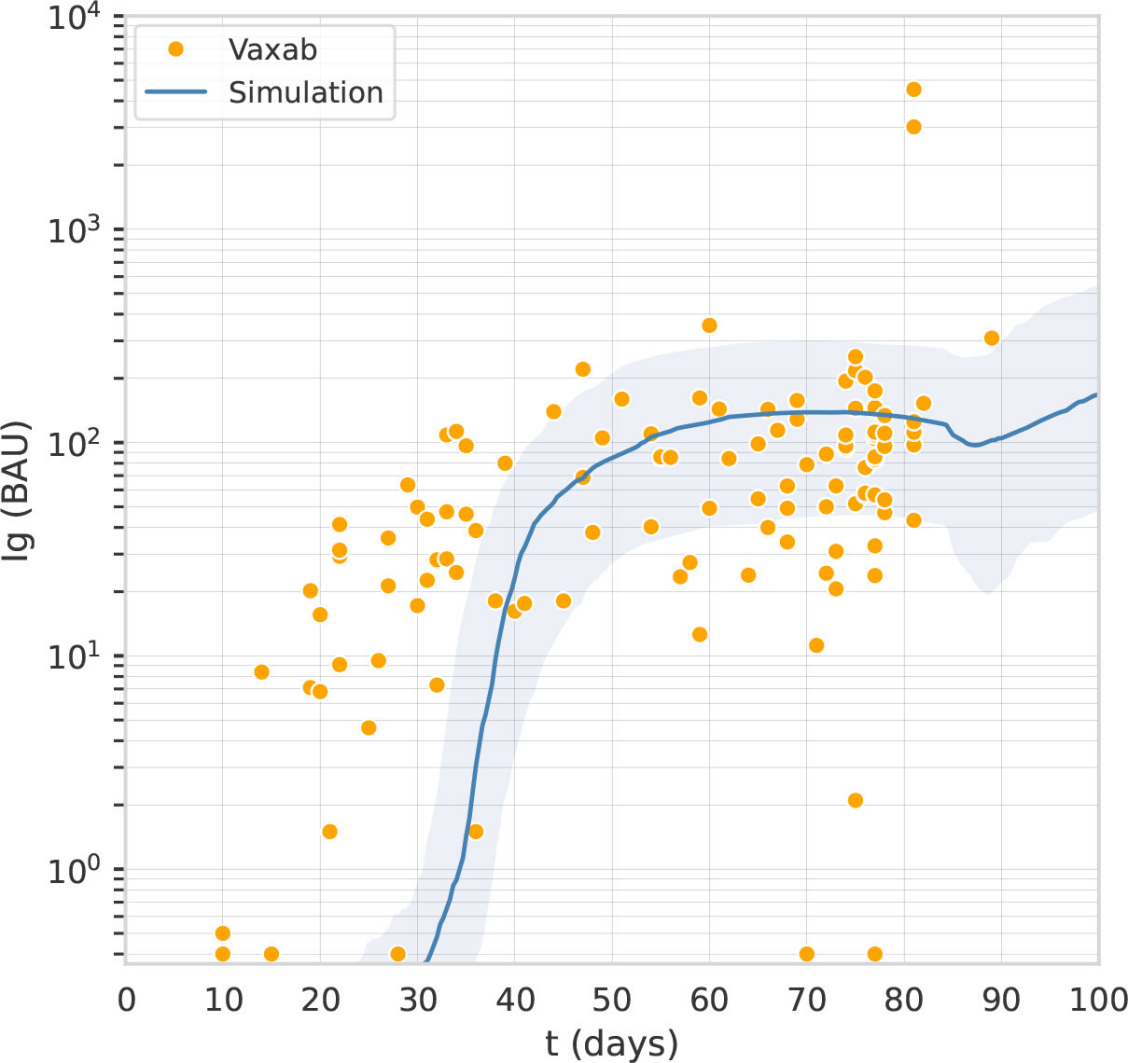
The computational model captures the antibody titer trajectory, characterized by a plateau. Overlay of a line graph representing in-silico Ig levels after the first dose (the line represents the median, the shading represents the IQR) with a dot plot representing RBD-Spike Ig BAU in individuals who have received one dose of AstraZeneca, in the Vaxab dataset.

Vaxab is an observational study of serological data in COVID-19 vaccinees. The study was approved by the Ethical Committee of the University of Naples Federico II, protocol 376/21. Inclusion criteria for participation were: i) having received a COVID-19 vaccine, ii) requesting a Roche Elecsys^®^ Anti-SARS-CoV-2 S assay at the MeriGen laboratory (Naples, Italy), iii) answering a questionnaire, and iv) signing the informed consent. Most study participants measured their antibody levels just once. Each dataset entry includes the age and sex of the participant, vaccination date(s), vaccine brand for each dose, the SARS-CoV-2 infection history (self-reported), the date of the serological test, and the result of the Roche Elecsys^®^ Anti-SARS-CoV-2 S assay, expressed in Binding Antibody Units (BAU). The Roche Elecsys^®^Anti-SARS-CoV-2S assay quantifies antibodies against the Receptor Binding Domain (RBD) of the S protein (the Spike) of SARS-CoV-2; the dynamic range can be scaled by automated sample dilution (23). The test was performed according to the manufacturer’s instructions (Roche Diagnostics GmbH. Elecsys^®^ Anti-SARS-Cov-2 s, Instructions for Use. 2021). The Vaxab study includes participants vaccinated with AstraZeneca, Pfizer, Moderna, and Johnson & Johnson vaccines. It contains 120 antibody measures from individuals immunized with one dose of AstraZeneca and who reported no previous SARS-CoV-2 infection. The age range of this subset of participants was 20-79 (median 53, IQR 38-61), with 62% females. The timing of blood tests was between 10 and 89 days after the first dose. Six individuals had no anti-Spike antibodies (baseline value of the test, 0.4 BAU), whereas two outliers (not visible in **Figure 1**) had anti-Spike BAU levels >10000. The data points of the Vaxab study (one dose of AstraZeneca, no previous SARS-CoV-2 infection) are shown in **Figure 1**.

### 2.2 Dataset 2: The trials COV001 and COV002

**Figure 2:**
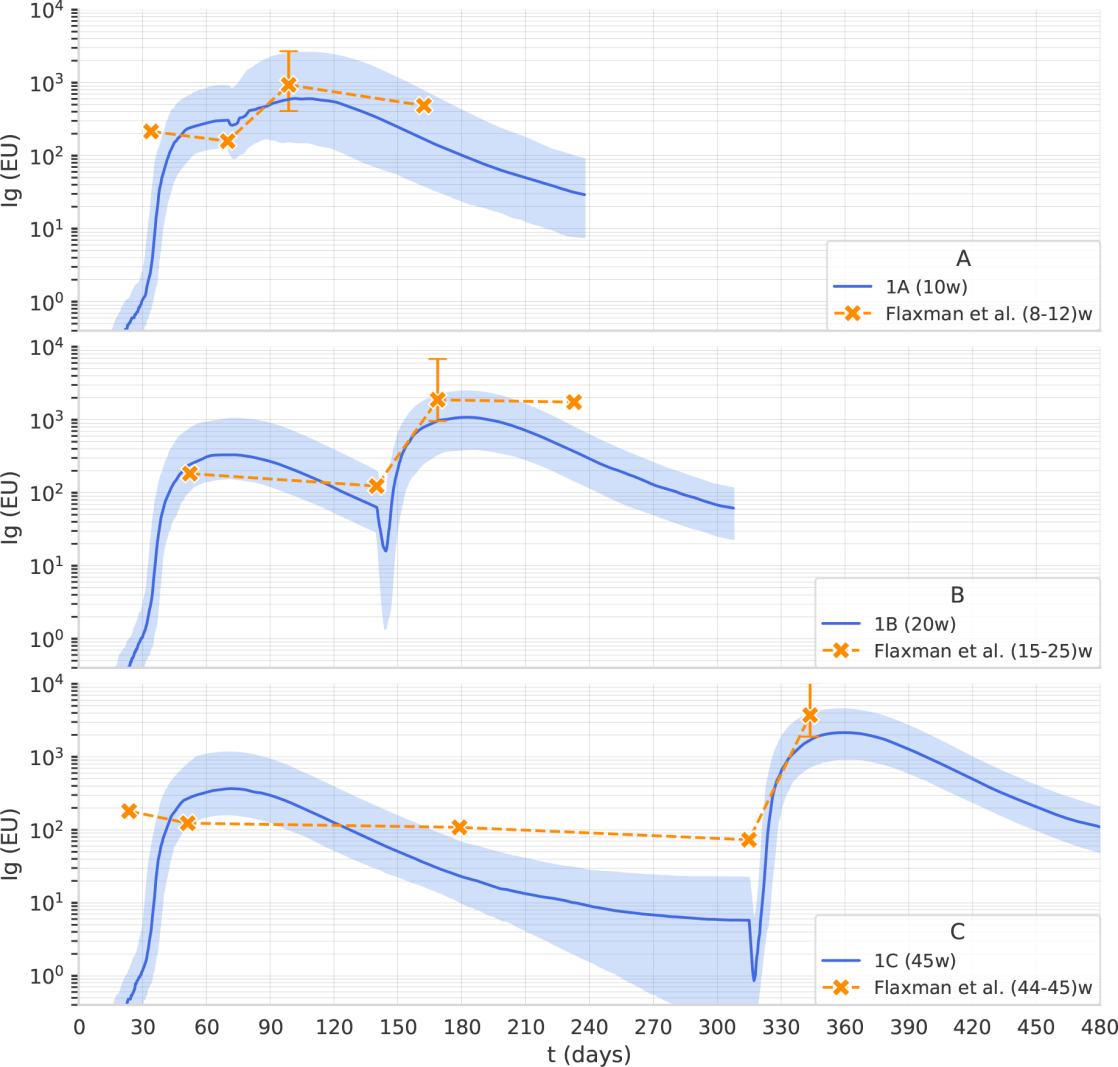
The computational model reproduces the effect of the dosing interval observed in clinical trials. Overlay of in-silico Ig levels in two-dose protocols 1A, 1B, and 1C (the line represents the median, the shading represents the IQR) with a dotplot representing median anti-Spike Elisa Units in clinical trial data from (11), corresponding to inter-dose periods of 8-12, 15-25 and 44-45 weeks for panel **(A–C)**, respectively.

Flaxman et al. (11) reported the immunogenicity of AstraZeneca with 3 different dosing intervals, namely 8–12, 15–25, and 44–45 weeks. Blood samples were taken on the day of vaccination and then at 14 and/or 28 days after vaccination. Antibody levels to SARS-CoV-2 Victoria/01/2020 spike were measured by standardized single dilution total IgG ELISA, and the median for each group was reported. On day 28 the median total IgG titer was 923 Elisa Units (EU) with Interquartile Range (IQR) [525–1764] for the 8–12 weeks interval, 1860 EU and IQR [917–4934] for the 15–25 weeks interval, and 3738 EU IQR [1824–6625] for the 44–45 weeks interval. Antibody levels 6 months after the second dose of vaccine were higher in the group with a 15–25 weeks interval between doses, median 1240 EU IQR [432–2002], compared with the group with 8–12 week interval, median 278 EU IQR [166–499]. Data points for this data set are shown in **Figure 2**.

### 2.3 Computational model

The computational model we used in this study has been previously employed to simulate the immune response to different antigens including SARS-CoV-2 virus (24). Most of the model parameters have already been fixed either by manual curation with literature information or by numerical estimation in general settings. For the current purpose, we modified this computational model to simulate the immune response to a non-replicative adenovirus carrying a transgene encoding the Spike protein of SARS-Cov-2, and then we adjusted the model’s parameters using data from human vaccination with Astra Zeneca. The model represents both the innate immune response by macrophages, dendritic cells, and natural killer cells and the adaptive immune response by B lymphocytes, antibody-producing plasma cells, CD4 T helper, and CD8 T cytotoxic lymphocytes. It is a polyclonal model as it embodies the primary sequences of the binding sites of B-cell receptors (BCR) and T-cell receptors (TCR), as well as the peptides and epitopes of the infectious agent or vaccine. It represents a portion of i) the muscle, where the vaccine is injected, ii) primary lymphatic organs where lymphocytes are formed and mature, and iii) secondary lymphoid organs where antigens are presented to naïve B and T-cells. Further details are provided in the **Supplementary Material**.

To evaluate different vaccination protocols we have used the antibody level as a significant endpoint representative of the immunogenicity of the vaccine construct. Neutralizing antibody levels are known to correlate with immunity from symptomatic SARS-CoV-2 infection (25, 26).

### 2.4 Parameters identification

The parameters of the immune system simulator were identified fitting two datasets introduced in *Dataset 1: The observational study Vaxab* and *Dataset 2: The trials COV001 and COV002*. The first round of manual calibration, based on the Vaxab dataset, aimed at reproducing the trajectory of the antibody levels after the first vaccine dose. The Vaxab dataset shows that, after the first dose of AstraZeneca, anti-Spike antibody levels increase for 5-6 weeks and then remain stable until the 12th week, when the second dose is received. This antibody titer trajectory, characterized by a plateau, was captured by acting on parameters related to vaccine dosage, antigen release kinetics from the adenovirus-transfected cells, and the scaling factor that adjusts the model scale to the antibody concentrations expressed in BAU (**Figure 1**).

The second fine-tuning step, using data from the published clinical trials described in *Dataset 2: The trials COV001 and COV002*, aimed to capture the effect of dosing interval on antibody titers. We used the Approximate Bayesian Calculation (details provided in **Supplementary Material**) to estimate some parameters, namely the persistence of phagocytosed antigen before it is degraded in the cytosol of APCs, the plasma-long-lived/normal half-life, and the rate of spike production from infected muscle cells. As shown in **Figure 2**, the computational model reproduces the effect of the dosing interval observed in clinical trials. Note that the scaling factor used in **Figure 2** is different from the one employed in **Figure 1** as it relates to antibody titers obtained with a different assay and expressed in EU rather than in BAU.

### 2.5 In-Silico experiments

We used the model to perform in-silico experiments of vaccination with two doses (in what we call experiment 1) or three doses (experiment 2) of adenoviral COVID-19 vaccine. Each experiment included multiple treatment groups, differing in the time interval between doses (**Table 1**). In particular, experiment 1 includes three treatment groups, denoted 1A, 1B, and 1C, that differ in the interval between the first and second dose, which is 10 weeks in protocol 1A, 20 in protocol 1B, and 45 in protocol 1C. While experiment 2 includes nine treatment groups, denoted 2A-I that differ in the interval between the second and third dose, which is 4 months in protocol 2A, 6 in protocol 2B, 8 in protocol 2C, 10 in protocol 2D, 12 in protocol 2E, 14 in protocol 2F, 16 in protocol 2G, 20 in protocol 2H and 24 months in protocol 2I. In all treatment groups of experiment 2, the second dose is given 12 weeks after the first dose. Each treatment group included 200 individuals and the follow-up was 6 months after the last dose.

**Table 1:** *In-silico* vaccination experiments.

	Vaccination regimen	Treatment group (dosing protocol)	Interval between 1^st^ and 2^nd^ dose	Interval between 2^nd^ and 3^rd^ dose	Follow-up
Experiment 1	Two doses	1A	10 weeks	–	6 months after 2nd dose
		1B	20 weeks	–	
		1C	45 weeks	–	
Experiment 2	Three doses	2A	12 weeks	4 months	6 months after 3rd dose
		2B	12 weeks	6 months	
		2C	12 weeks	8 months	
		2D	12 weeks	10 months	
		2E	12 weeks	12 months	
		2F	12 weeks	14 months	
		2G	12 weeks	16 months	
		2H	12 weeks	20 months	
		2I	12 weeks	24 months	

### 2.6 Statistical analysis

The statistical analysis aimed at i) establishing whether the immune response is statistically different among the protocols detailed in *In-silico experiments*, ii) identifying the right timing for the third dose and iii) investigating whether the antibody response is linked to some immunological variable. To this end we focused our analysis on five components of the immune system, that play a critical role in vaccine efficacy, namely antibodies (*i.e.*, the sum of IgG1and IgG2), denoted by *Ab*, plasma cells, denoted by *Plb*, memory T helper cells, denoted by *Th*, memory T cytotoxic cells, denoted by *Tc*, and memory B cells, denoted by *B*.

The dynamics of these variables is shown in **Figure 4**.

**Figure 3:**
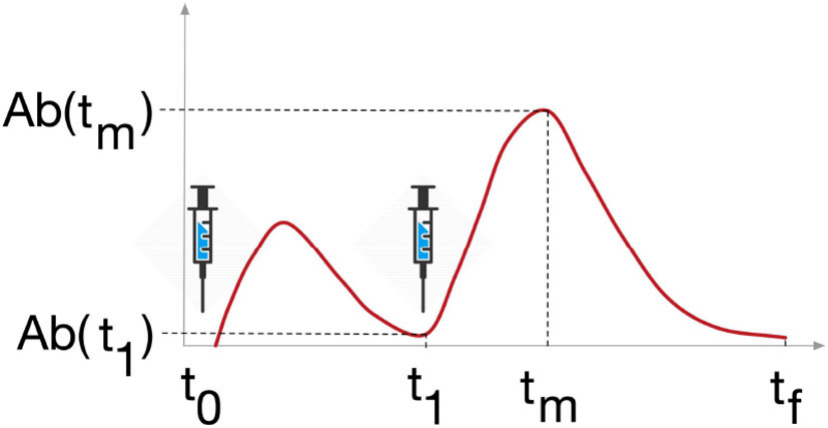
Study design. Variables Ab, Plb, Th, Tc and B were analyzed at 3 timepoints, t_1_ is the timepoint before the last dose of vaccine, t_m_ is the timepoint when the variable reaches its maximum after the last dose, and t_f_ is the last timepoint of the simulation, 6 months after the last dose.

To fulfill the tasks detailed above, we analyzed the variables of interest at crucial time points, namely the time before the second dose, denoted by *t*_1_, the time when the variable reaches its peak after the second dose, denoted by *t_m_*, and the latest time point of the simulation six months after the second dose, denoted by t_f_ (**Figure 3**.)

First, for each treatment group (**Table 1**), we report standard sample statistics such as median, IQR, minimum and maximum. Mann-Whitney test is used to asses significant differences in the variables of interest at *t*_1,_ *t_m_* and *t_f_* among the different treatment group, task i), and, in particular, differences in

*Ab*(*t_m_*) is used to asses the optimal timingfor the third dose, task ii). Stepwise regression (explained in detail in **Supplementary Material**) is used to determine whether variables *Ab*(*t*_1_), *Plb*(*t*_1_), *Th*(*t*_1_), *Tc*(*t*_1_), and *B*(*t*_1_) can be used as explanatory variables for the increment of *Ab* induced by the second dose, that is *Δ*_*Ab*_= (*Ab*(*t*_*m*_) –*Ab*(*t*_1_), task iii). Then, correlations and cross-correlations at *t*_1,_ *t_m_* and *t_f_* (**Figure 3**) between the variables of interest are investigated in terms of Pearson’s correlation coefficient. Finally, given the results of correlations analysis, Principal Component Analysis and Principal Component Regression (explained in detail in **Supplementary Material**) are employed to better investigate the link between the antibody response and the other immunological variables.

Moreover, to test whether in-silico experiments show patterns that can be traced to immunological behavior of interest, we performed unsupervised clustering on *Ab*(*t*_1_) Specifically, we applied a machine learning method, k-means clustering, which partitions N observations into K groups such that the within-cluster variance is minimal, see (27) for details.

## 3 Results

### 3.1 The interval between doses affects immunological memory

**Figure 4:**
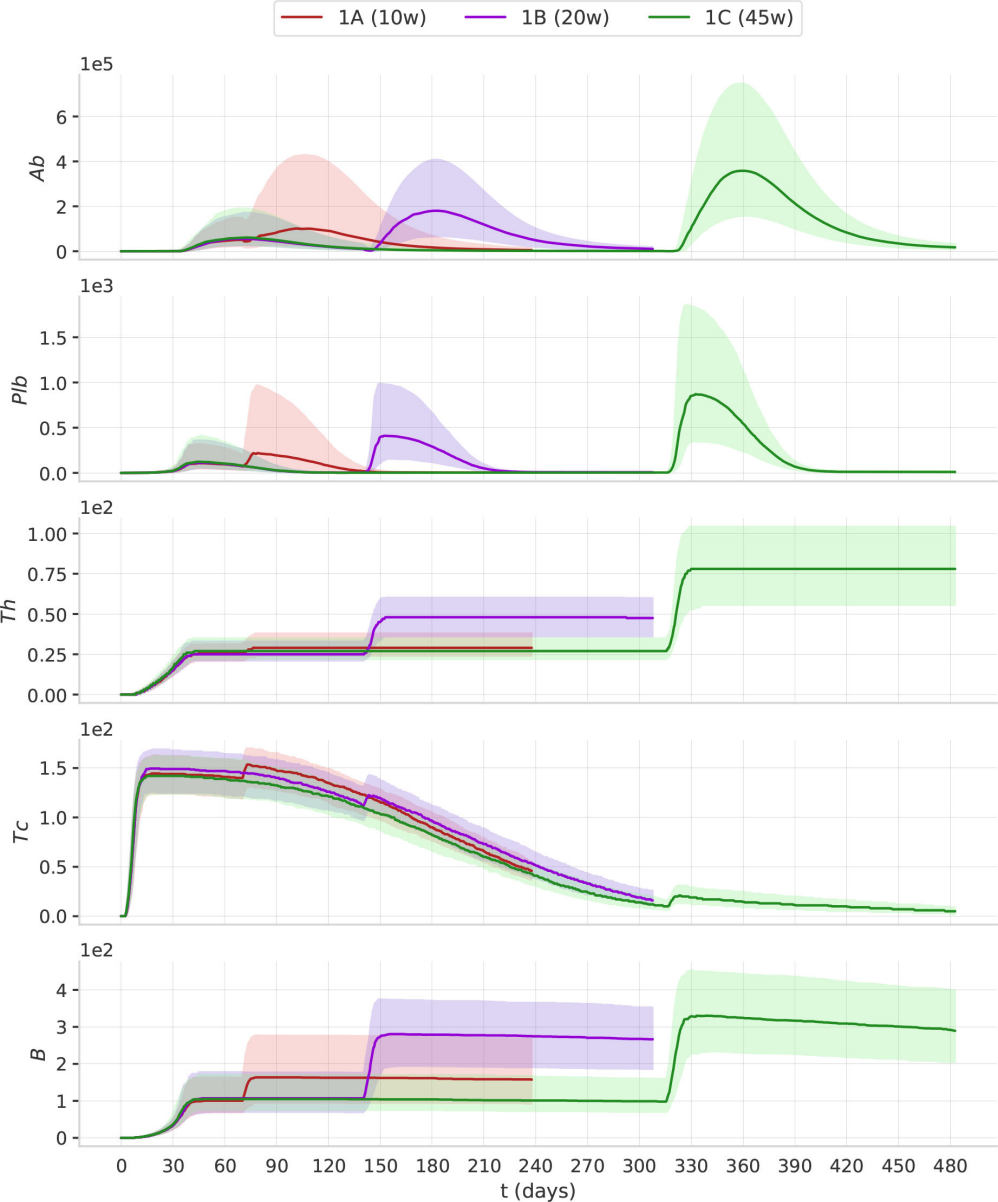
The timing of the second dose affects the dynamics of the immune response. The plots represent the median (solid lines) and IQR (shaded area) of *Ab, Plb, Th, Tc, B.* Protocols with longer intervals between the first and second dose achieve higher antibody responses.

In **Figure 4**, we report the dynamics of *Ab*, *Plb*, *Th*, *Tc* and *B* in the treatment groups 1A, 1B, and 1C. The second dose of the vaccine induces a peak of plasma cells, higher than the peak induced by the first dose. Plasma cell peaks are mirrored by antibody peaks. *Th* and *B* after the second dose reach a higher level, and their increase persists for the following 6 months of simulation. When we compare 1A, 1B, and 1C, we see that as the interval between the two doses becomes longer, the humoral response (*i.e.*, *Ab*, *Pbl*, *B*) and the T helper response (*Th*) to the second dose improve. This advantage of the longer protocols is still evident 6 months after the second dose (**Supplementary Material Figure S3**). Interestingly, the trajectory of *Tc* is markedly different from all other trajectories. The first dose has the major effect on *Tc* expansion, not the second (**Figure 4**). This finding agrees with studies reporting the absence of a significant boost of the cellular response after the second ChAdOx1 nCoV-19 dose (13).

### 3.2 The antibody response to the second dose correlates with the number of pre-existing memory B cells and is mitigated by pre-existing cytotoxic T cells and antibodies

**Figure 5:**
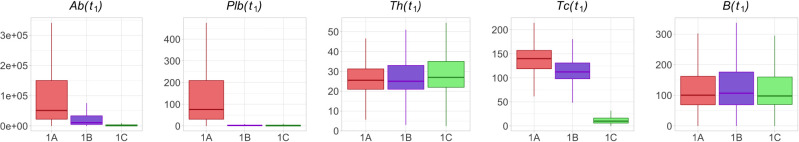
Th and B are not significantly different in protocols 1A, 1B and 1C at time t_1_, whereas Ab, Plb and Tc are lower in the longer protocols. The box plots show the median, IQR, and range of Ab, Plb, Th, Tc and B in treatment groups 1A, 1B and 1C at time t_1_.

Protocols with longer inter-dose intervals induce higher *Ab*, *Plb*, *Th*, and *B* (**Figure 4**). To shed light on the immunological mechanisms underlying this phenomenon, we set out to analyze how the immune status at *t*_1_ (*i.e.*, before vaccination) affects the subsequent antibody increment Δ*_Ab_*, that is the difference between the peak value *Ab*(*t_m_*) and the pre-existing antibody level *Ab*(*t*_1_). A stepwise regression analysis indicates that, within each protocol 1A, 1B and 1C, pre-existing memory B cells B(*t*_1_) is the only variable which significantly influence Δ*Ab*. *B*(*t*_1_) is not significantly different between protocols 1A, 1B, and 1C (**Figure 5**), therefore, the differences in the magnitude of the antibody response to the second dose between the shorter and longer protocols cannot be imputed to memory B cells. Instead, both *Ab*(*t*_1_) and *Tc*(*t*_1_) are significantly lower in the longer protocols (**Figure 5**). This supports the hypothesis that either *Ab*(*t*_1_) or *Tc*(*t*_1_), or both, may have an inhibitory effect on the antibody response to the second dose.

**Figure 6:**
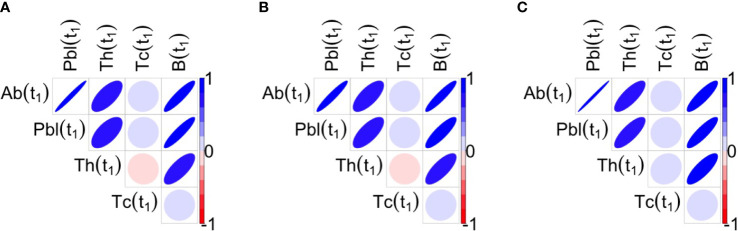
Correlations between the variables of interest at t_1_ for the three protocols 1A, 1B, 1C are shown respectively in panels **(A–C)**. Blue ellipses mean positive correlations while red ellipses mean negative correlations, as reported in the color bar. The shape of ellipse helps in the understanding: the more stretched the ellipse the higher the value of the correlation in absolute value. At t_1_, antibodies, plasma cells, memory B cells and memory T helper cells are positively correlated among them, whereas Tc is not significantly correlated with the Other variables.

However, by exploring the correlations and cross-correlations among variables *Ab*, *Plb*, *Th*, *Tc* and *B*, it was found that at *t*_1_, in all three protocols, antibodies, plasma cells, memory B cells and memory T helper cells were positively correlated with each other, whereas memory T cytotoxic cells were not significantly correlated with the other variables (**Figure 6**). Note that significant correlations among variables may influence the results of the stepwise regression.

**Figure 7:**
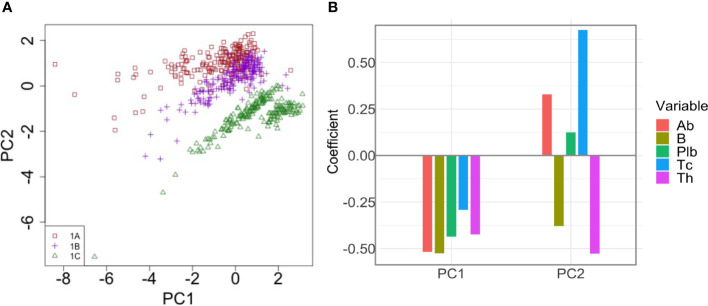
Principal Component Analysis of the correlation between pre-existing immunological memory at t_1_ and the peak value of the antibody response to the second dose. **(A)** The dot plot shows PC1 and PC2 in individuals in treatment groups 1A, 1B and 1C. PC2 separates the different dosing protocols. **(B)** Loadings of PC1 and PC2. In PC2, Tc has the highest loading.

To better understand how the interplay between the variables of interest in *t*_1_ contributes to the enhanced antibody response to the second dose after longer intervals, we performed a Principal Component Regression between *Ab*(*t*_1_), *Plb*(*t*_1_), *Th*(*t*_1_), *Tc*(*t*_1_), *B*(*t*_1_) and the peak value of the antibody response to the second dose, *Ab*(*t_m_*). We obtain five principal components, PC1-5, that explain 45% of the variance of *Ab*(*t_m_*). Of these, PC1 and PC2 are the two most important components, and together explain 38.54% of the variance of *Ab*(*t_m_*). **Figure 7** shows a scatterplot of PC1 vs PC2. Each dot represent one simulation, *i.e.*, one virtual individual. Protocols 1A, 1B and 1C form separate clusters. At *t*_1_, 1A, 1B and 1C are similar in PC1, and are separated by PC2, suggesting that PC1 explains differences in *Ab*(*t_m_*) among individuals who received the same dosing protocol, whereas PC2 is more relevant to understand the difference between the 1A, 1B and 1C, *i.e.*, the effect of the timing of the second dose on *Ab*(*t_m_*). Interestingly, the 1C group separates into two distinct clusters that are different in PC1. The most important variables (highest coefficient or loading) within PC1 are *B*(*t*_1_) (loading -0.525), and *Ab*(*t*_1_) (loading -0.519)(**Figure 7**). So PC1 mainly represents pre-existing antibody levels and B cell memory, which are positively correlated to Ab(*t_m_*), irrespective of the timing of the second dose. The highest loadings within PC2 are *Tc*(*t*_1_) (0.675) and *Th*(*t*_1_) (-0.527) (**Figure 7**), therefore PC2 mainly represents T cells. Notably, memory T cytotoxic cells and memory T helper cells exert opposite effects (*i.e.*, opposite sign of the loading coefficients). Overall, the PCA suggests that pre-existing memory T cytotoxic cells are the major correlates of the reduced immunogenicity that is observed when the second dose is given at earlier time points. In PC2, the pre-existing antibodies also display the same sign of the coefficient as memory T cytotoxic cells, yet with a lower absolute value.

### 3.3 The Tc response to the second dose of vaccine is limited by the number of antigen presenting cells

The increase of *Tc* after the second dose is much smaller than the increase of *Tc* after the first dose (**Figure 4**). In order to proliferate, *Tc* need to recognize their cognate epitopes complexed with MHC class I on the vaccine-transduced muscle cells. Hence, to understand what may cause the poor response of *Tc* to the second dose, we analyzed antigen presentation on MHC class I, in muscle cells, after each dose. To estimate the total amount of antigen presentation on MHC class I that occurs after the first and second dose of vaccine, we calculated the Area Under the Curve (AUC) of the model variable “class-I-presenting” ( $\text{AUC=}\int_{t_{1}}^{t_{f}}m\;(t)dt$ where *m*(*t*) is the number of antigen presenting cells, which indicates the cumulative number of muscle cells that present vaccine antigen peptides on MHC class I, in the time interval [*t*_1_,*t_f_*]). The AUC of the first dose was calculated from *t*_0_ to *t*_1_, while the AUC of the second dose was calculated from *t*_1_ to *t_f_*. **Table 2** reports the median and interquartile range of AUC, in protocols 1A, 1B and 1C. Antigen presentation on MHC class I on muscle cells after the second dose of vaccine is lower than after the first dose, which explains why *Tc* are poorly stimulated by the second dose (see **Table 2**).

**Table 2:** Antigen presentation on MHC class I in muscle cells.

	AUC MHC class-I-presenting	
	First dose (*t*_0_ to *t*_1_)	Second dose (*t*_1_ to *t*_f_)
1A	7904 (6314.50-9971)	114.5 (82.75-154.50)
1B	8002 (6604.25-10117)	272.5 (177.24-366.25)
1C	7569 (6125.50-9451)	540.5 (309.75-871)

### 3.4 The optimal antibody response to the third dose is achieved over a large time window

**Figure 8:**
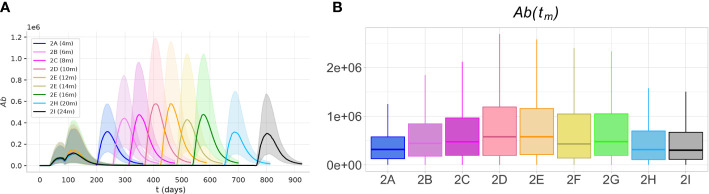
The optimal immunogenicity of antibody response to the third dose is achieved over a large time window. The protocols with intervals between the second and third dose between 6 and 16 months achieve peak antibody responses significantly higher (*p*< 0.05)) than shorter or longer protocols. **(A)** The plots represent the dynamics of the median (solid lines) and the IQR (shaded area) of variable *Ab*, in experiments 2A-I. **(B)** The box plots show the median, IQR, and range of the antibody peak after the third dose, *Ab*(*t_m_*).

To predict the optimal timing for the third dose, we analyzed the results of experiment 2. The simulations predict that the optimal antibody response to the third, booster dose, is achieved over a large time window, spanning from 6 to 16 months after the second dose (**Figure 8**). Over this time window, the peak antibody levels are significantly higher (*p*< 0.05, Mann-Whitney test) than those achieved with earlier or later boosters.

Presumably, in the first months after the second dose, the high levels of antibodies and cytotoxic T cells inhibit the response to the third dose. On the other hand, much later after the second dose, when the memory Th and B cells and long-lived plasma cells decline, the antibody response to the third dose is reduced.

### 3.5 At late timepoints individual responses form two clusters with different antibody dynamics, sustainers and decayers

**Figure 9:**
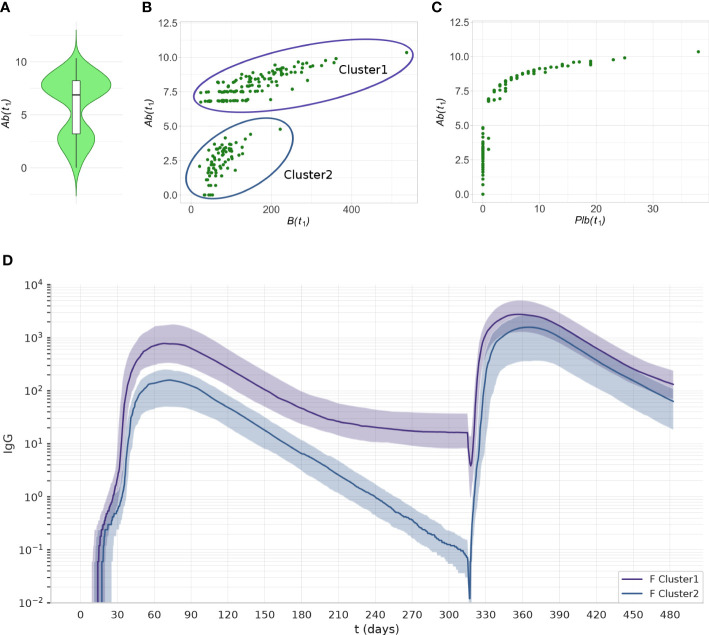
The two clusters identified by PCA can be separated by their level of *Ab*(*t*_1_) Data from experiment 1C are reported. **(A)** The violin plot of *Ab*(*t*_1_) reveals two clusters with different levels of Ab. **(B)** The scatterplot shows that the individuals with low antibody levels have, in most cases, no plasma cells. **(C)** the scatterplot shows that the individuals with low levels of antibody have memory B cells. **(D)** The antibody dynamics of the two clusters is different, cluster 1 represents antibody *sustainers*, and cluster 2 represents antibody *decayers*. The plots represent the median (lines) and IQR (shaded area) of variable *Ab* in cluster 1 and cluster 2.

Interestingly, the principal component analysis revealed two separate clusters in the experiment 1C at *t*_1_. The level of *Ab*(*t*_1_) allows separation of the two clusters (**Figure 9A**). The cluster with the lower level of antibodies has memory B cells (**Figure 9B**), but no plasma cells (**Figure 9C**). The number of memory B cells and memory T helper cells at *t*_1_ is significantly different between cluster 1 and cluster 2 (p< 10^-7^).

We analyzed the antibody dynamics in the two clusters (**Figure 9D**). Once the peak of antibodies generated by the first dose of vaccine has declined, individuals from cluster 1 (antibody *sustainers*) reach a plateau in their antibody levels that reflects the production by long-lived plasma cells. In contrast, individuals in cluster 2 (antibody *decayers*), have no long-lived plasma cells, therefore the decline of their antibody levels continues. These two patterns in the antibody trajectories result in the bimodal distribution of antibody levels in the population, at late timepoints after the last dose (**Figure 9A**).

**Figure 10:**
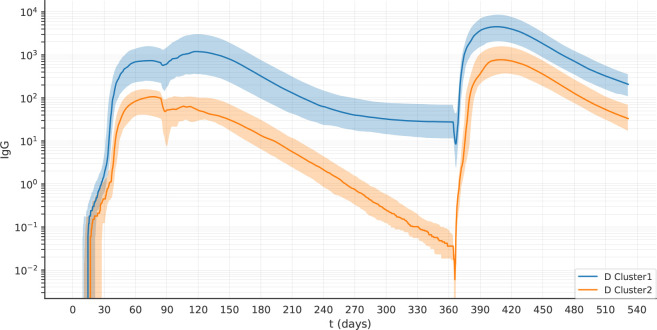
After two doses of vaccine, the virtual individuals can still be separated into clusters 1 and 2, representing antibody *sustainers* and *decayers*. The plots represent the median (lines) and IQR (shaded area) of variable Ab in clusters 1 and 2 in experiment 2D.

We observed *decayers* both after one dose of vaccine (**Figure 9**), and after two doses (**Figure 10**). The frequency of *decayers* however was lower after two doses (36.5% after 2 doses vs 20% after 3 doses). This suggests that as multiple doses of vaccine are administered, the number of individuals that will lose their serological immunity over time is reduced.

In experiment 1C, we identified the *decayers* looking at *t*_1_, namely 45 weeks after the first dose. We asked if, by machine learning clustering on antibody titers, *decayers* could be reliably identified at earlier time points. Therefore, to identify the optimal time to detect *decayers*, we performed k-means clustering at different weeks from the first dose and we compared the performance in terms of accuracy (*i.e.*,(*TP*+*TN*)/*N* where TP=true positive, TN=true negative, N=total population).

It turned out that from 28 weeks after the first dose, clustering on antibody titers allows the identification of antibody *decayers* with accuracy above 90% (**Table 3**).

**Table 3:** Accuracy of the identification of decayers.

Weeks							
	20	24	28	32	36	40	45
Accuracy	0.805	0.840	0.925	0.975	0.985	0.990	1

## 4 Discussion and conclusions

In the context of the COVID-19 pandemic, vaccination policies had to take into account vaccine supply constraints and disease burden. In some cases, due to vaccine supply shortage, the second dose was delayed to allow for a higher initial coverage with one dose. On the other hand, in some countries, in situations of high dose availability, a third dose of COVID-19 vaccine has been offered to the general population as early as 4 months after the second dose, to try and mitigate large infection waves driven by virus variants. The time delay between vaccine doses can affect the durability of the antibody response, as well as the probability of an enhanced response to a subsequent encounter with the same antigen (28–30).

We explored, in-silico, the optimal timing for the third dose of an adenoviral vaccine. We adjusted the model parameters using two sets of anti-Spike antibody measures obtained with two different assays. While different antibody assays can show different kinetics (31) because some assays are tuned for high-avidity antibodies (32), the antibody assays employed in the two datasets give a similar kinetics after the first dose of AstraZeneca, i.e., no major variation in the antibody titer between 4 and 12 weeks. For this reason, in **Figures 1**, **2** we could simply use two different scaling factors to adjust the scale of the model output to the antibody concentrations obtained with the two different assays.

Simulations predict that the optimal immunogenicity of the third dose is achieved over a large time window, spanning from 6 to 16 months after the second dose. We analyzed the contribution of pre-existing antibodies, plasma cells, memory B cells, memory CD8 T cells, and memory CD4 T cells on immunogenicity, in vaccination schedules with different intervals between the first and second dose, namely 10, 20, and 45weeks. We observe a strong positive correlation between antibodies, plasma cells, memory B cells, and memory CD4 T cells after the first dose of vaccine. It is important to underline that these strong correlations complicate the identification of the causal correlates of the effect of the timing of the second dose on immunogenicity. On the other hand, these correlations imply that the antibody titer, an element that can be very easily measured, is a biomarker of the numbers of memory B cells, plasma cells, and CD4 T cells.

In-silico, we allowed the antibodies induced by the first dose of vaccine to inhibit the entry of the adenoviral vaccine into cells, to reproduce the effect of antibodies directed against the adenoviral capsid. In clinical trials, anti-vector neutralizing antibodies have been detected after the first dose of adenoviral vaccine, but no correlation was observed between pre-existing anti-vector neutralizing antibodies and the response to the second dose. Also in our in-silico analysis, the mere analysis of the correlation between the pre-existing antibody titer within one experiment and the subsequent response does not reveal a negative correlation. The potential inhibitory effect of antibodies is revealed by the comparison between simulations in which antibodies were or were not allowed to neutralize the adenoviral entry in muscle cells (**Supplementary Material Figure S2**). Interestingly, longer intervals resulted in higher immunogenicity in both scenarios, therefore irrespective of the action of neutralizing anti-vector antibodies. In this context, we should emphasize that cytotoxic T cells against the Spike, a desired outcome of immunization, can contribute to reduced immunogenicity of subsequent doses.

The scenario that emerged from our in-silico analysis is that memory B cells and memory CD8 T cells have opposite effects on the antibody response to the boost. Increased antibody response to late booster doses appears to be due to the combined effect of the decline in antibody levels and in the number of memory CD8 T cells, which results in a higher amount of Spike antigens being produced.

In the simulations, the number of memory B cells is similar at 12, 20 and 45 weeks after the first dose of the vaccine. This prediction is in line with the long persistence observed after Sars-CoV-2 infection (33, 34), and in mouse studies of B cell memory (35).

Garg et al. previously analyzed the effect of prime-boost interval on the antibody response to vaccination in a stochastic simulation model of the germinal center reaction, and concluded that increased B cell selection stringency in the germinal center can explain improved COVID-19 vaccine efficacy with delayed boost (36). Affinity maturation is implemented in our model; however, we did not measure the effect of dose interval on affinity due to computational constraints. On the other hand, our model considers the impact of pre-existing antibodies, plasma cells, memory T helper cells and memory T cytotoxic on the magnitude of the response to the boost, and we show a major contribution of cytotoxic T cells and antibodies.

An interesting observation coming from the principal component analysis is that the individuals who underwent a longer inter dosage vaccination schedule separated into two distinct clusters. The two clusters contain, respectively, individuals that generated or did not generate long-lived plasma cells. The dynamics of the antibody titers is markedly different between the two groups: one group reaches a plateau of antibody levels (*sustainers*), while the other group is destined to sero-revert (*decayers*). In this in-silico system, these qualitative differences stem from stochastic inter-individual differences in the immune repertoire and the efficacy of priming. We speculate that, in real life, aged and immunocompromised people may be prone to the decayer pattern and may benefit from receiving their booster after a shorter interval. Indeed, after two doses of adenoviral vaccine, waning of vaccine effectiveness against symptomatic COVID-19 is greater in older adults and in those in a clinical risk group (37). Machine learning clustering on antibody titers allows the identification of the *decayers* with 0.925 accuracy as early as 28 weeks after the first dose.

Our observation of two subsets of vaccinated individuals with different antibody dynamics over time resembles the observation of two subsets of COVID-19 convalescents, antibody *sustainers*, that exhibited the same or increasing antibody levels over time, and antibody *decayers* that lost antibody levels over the same time frame (38). A tetramer-based analysis of T follicular helper cells suggested a connection between Spike-specific CD4+ T cell responses and anti-Spike antibody durability (39). In addition, more memory B cell cross-reactivity with endemic coronaviruses was identified as a marker for more sustained antibody responses after infection (40). Our in-silico experiments replicate these correlations, as also in the simulation antibody *sustainers* have more memory T helper and memory B cells than antibody *decayers*. Our analysis suggests that a stronger response of T helper and B cells has a higher probability of resulting in the development of long-lived plasma cells.

Following asymptomatic or pauci-symptomatic SARS-CoV-2 infection, higher peak anti-Spike responses have been associated with longer time to sero-reversion (41). Our in-silico experiments replicate this correlation, as also in the simulations antibody *decayers* have a lower anti-Spike peak than antibody *sustainers*.

Neutralizing antibodies against the SARS-CoV-2 Spike are known to correlate with immunity from symptomatic infection, therefore unraveling the long-term kinetics of antibodies after SARS-CoV-2 infection or COVID-19 vaccination, and the factors influencing it, is essential to optimize vaccine boosting strategies (25, 26). Our analysis suggests that while the time window for the optimal immunogenicity of the third dose of an adenoviral vaccine is ample (6-16 months), however, some individuals, namely the antibody *decayers*, may benefit from receiving the third dose at the beginning of the optimal time window, to avoid loss of serological protection.

## Data availability statement

The raw data supporting the conclusions of this article will be made available by the authors, without undue reservation.

## Ethics statement

The studies involving human participants were reviewed and approved by Ethical Committee of the University of Naples Federico II, protocol 376/21. The patients/participants provided their written informed consent to participate in this study.

## Author contributions

FC and AP contributed to the conception and design of the study. IDB and GP collected informed consents from study participants. IDB, SDB, GP, and AP organized the database. FC and EM performed the in-silico experiments. PS performed the statistical analysis. AP wrote the first draft of the manuscript. AP, PS, FC, and EM contributed to data interpretation. FC, PS, and EM wrote sections of the manuscript. All authors contributed to manuscript revision, read, and approved the submitted version.

## Funding

FC and PS wish to thank the Italian Ministry of Education, University and Research, for partial support under the frame of JPI AMR (project MAGIcIAN, N. 0000873).

## Acknowledgments

The investigators express their gratitude for the contribution of the Vaxab participants. AP acknowledges Mariarosaria Aletta for bibliographic assistance.

## Conflict of interest

Authors IDB and SDB were employed by company MeriGen Res.

The remaining authors declare that the research was conducted in the absence of any commercial or financial relationships that could be construed as a potential conflict of interest.

## Publisher’s note

All claims expressed in this article are solely those of the authors and do not necessarily represent those of their affiliated organizations, or those of the publisher, the editors and the reviewers. Any product that may be evaluated in this article, or claim that may be made by its manufacturer, is not guaranteed or endorsed by the publisher.
