# Supplementary material for: In-silico evaluation of adenoviral COVID-19 vaccination protocols: Assessment of immunological memory up to 6 months after the third dose
Source: Front Immunol. 2022 Oct 24;13:998262. doi: 10.3389/fimmu.2022.998262 (PMC9639861; doi:10.3389/fimmu.2022.998262)
Supplement: Supplementary file 1 [file DataSheet_1.pdf]

Supplementary Material

1 THE COMPUTATIONAL MODEL

C-IMMSIM is a model that uses the concept of agents (*i.e.,* it is an agent-based model, ABM) to represent cells in the immune system [6]. A large number of cells are simulated in a virtual volume. When confronted with an antigenic challenge, their interaction/cooperation, governed by the rules describing their behaviour, evolve the system from a naive state to the state of immunological memory.

Being more a general-purpose modeling platform rather than just a model, C-IMMSIM lends itself to characterize the role of the immune response in different human pathologies ranging from infections to cancer passing through the chronic inflammation associated with type 2 diabetes ([8]). Relevant to the present study, the model has recently been used to simulate the clonal dominance in heterologous immune responses [8] and the response to a multi-epitope vaccine against SARS-CoV-2 [16, 1].

The system is in a stable state (apart from random fluctuations due to natural cell death/birth of cells) until the adenovirus vaccine is injected. This is day 0 of the simulation. It follows a sequence of stochastic events promoting cell duplication, cytokine secretion and eventually culminating in the humoral and cellular immune response. Due to the high degree of details of the algorithms enacting such events, an agent-based model is not described by means of mathematical formulas but logical rules or more complex algorithms that singularly or in combination with other rules, encode what are regarded as established mechanisms of the immune system (e.g., the clonal selection theory of Burnet, the thymus education of T lymphocytes, the replicative senescence of T-cells, T-cell anergy and Ag-dose induced tolerance in B-cells, and the danger theory of Matzinger).

In C-IMMSIM each simulated time step corresponds to eight hours of real life. Cells diffuse randomly in the represented volume and interact among them. Upon specific recognition through receptor bindings, they perform actions that determine their functional behavior. These actions are coded as probabilistic rules and define the transition of the interacting cell entities from one “condition” to another. In fact, each rule is executed only if the parts involved are in specific states (e.g., naïve, active, resting, antigen-presenting).

Besides cell-cell interaction and cooperation, this model simulates the intra-cellular processes of antigen uptake and presentation. Endogenous antigens are fragmented and combined with MHC class I molecules for presentation on the cell surface to CTLs’ receptors (this is the cytosolic pathway), whereas exogenous antigens are degraded into small pieces, which are then bound to MHC class II molecules for presentation to T helpers’ receptors (this is the endocytic pathway).

The stochastic execution of the algorithms coding for the dynamical rules results in a sequence of cause/effect events culminating in the production of effector immune cells and in the establishment of immunological memory. The starting point of this series of events is the injection of the adenovirus coding for the Spike protein of SARS-CoV-2 (see Figure S1). This may take place any time after the simulation starts. In the present study, the first immunization injection is performed at time zero. At that initial time, the system is “naïve” with respect to the injected antigen, meaning that there are neither specific T and B memory cells nor plasma cells and antibodies able to recognize the antigen peptides. Moreover, the system is designed to maintain the global population of cells in a quasi steady-state (homeostasis), if no stimulation takes place, whereas the system moves away from such dynamical (stochastic) equilibrium when it is perturbed by an antigenic challenge. Besides the parameters defining the characteristics of the adenovirus

related to entry in the muscle cells and production of the spike protein of the Severe Acute Respiratory Syndrome Coronavirus 2 isolate Wuhan-Hu-1 (NCBI Reference Sequence: NC_045512), whose primary structure is reported in Figure S1 and further described in the following section "Computing peptides immunogenicity", the coded vaccine construct in this model is defined as a set of B-cell epitopes and T-cell epitopes consisting of amino acid sequences and defining its antigenicity.

If the vaccine elicits a strong immune response it depends on the injected dose and on the antigenicity of the B and T epitopes. These variables determine the level deployment of both cellular and humoral branches of the immune system, as shown in past simulation studies [17]. The model resorts to pre-computed ranked lists of T-cell epitopes calculated with the neural network NetMHCpan method [18, 22, 21]. This feature, which is described below, follows the choice of a definite HLA set (discussed below in the section "Selecting the HLA haplotype"). As the neutralizing antibody response to the Spike protein is mainly focused on the RBD, and one of the datasets used to tune the model contains anti-RBD antibody measures, we included in the simulations only B-cell epitopes from the RBD domain. B-cell epitopes were computed with BepiPred [15]. The computed epitopes largely overlap with RBD regions experimentally identified as targets of anti-Spike antibody responses [12].

>YP_009724390.1 surface glycoprotein [Severe acute respiratory syndrome coronavirus 2]
MFVFLVLLPLVSSQCVNLTTRTQLPPAYTNSFTRGVYYPDKVFRSSVLHSTQDLFLPFFSNVTWFHAIHV
SGTNGTKRFDNPVLPFNDGVYFASTEKSNIIRGWIFGTTLDSKTQSLLIVNNATNVVIKVCEFQFCNDPF
LGVYYHKNNKSWMESEFRVYSSANNCTFEYVSQPFLMDLEGKQGNFKNLREFVFKNIDGYFKIYSKHTPI
NLVRDLPQGFSALEPLVDLPIGINITRFQTLLALHRSYLTPGDSSSGWTAGAAAYYVGYLQPRTFLLKYN
ENGTITDAVDCALDPLSETKCTLKSFTVEKGIYQTSNFRVQPTESIVR**FPNI**TNLCPFGEVFN**ATRFAS**V
YAWNRKRISNCVADYSVLYNS**ASFSTFK**CYGVSPTKLNDLCFTNVYADSFV**IRGDEVRQIAPGQTGKIAD**
YNYKLPDDFTGCVIAWNS**NNLDSKVGGNYNYLYRLFRKSNLKPFERD**I**STEIYQAGSTPCNGVEGFNCYF**
PLQSYGFQPTNGVGYQPYRVVVLSFELL**HAPATVCGPKKSTNLVKNKCV**NFNFNGLTGTGVLTESNKKFL
PFQQFGRDIADTTDAVRDPQTLEILDITPCSFGGVSVITPGTNTSNQVAVLYQDVNCTEVPVAIHADQLT
PTWRVYSTGSNVFQTRAGCLIGAEHVNNSYECDIPIGAGICASYQTQTNSPRRARSVASQSIIAYTMSLG
AENSVAYSNNSIAIPTNFTISVTTEILPVSMTKTSVDCTMYICGDSTECSNLLLQYGSFCTQLNRALTGI
AVEQDKNTQEVFAQVKQIYKTPPIKDFGGFNFSQILPDPSKPSKRSFIEDLLFNKVTLADAGFIKQYGDC
LGDIAARDLICAQKFNGLTVLPPLLTDEMIAQYTSALLAGTITSGWTFGAGAALQIPFAMQMAYRFNGIG
VTQNVLYENQKLIANQFNSAIGKIQDSLSSTASALGKLQDVVNQNAQALNTLVKQLSSNFGAISSVLNDI
LSRLDKVEAEVQIDRLITGRLQSLQTYVTQQLIRAAEIRASANLAATKMSECVLGQSKRVDFCGKGYHLM
SFPQSAPHGVVFLHVTYVPAQEKNFTTAPAICHDGKAHFPREGVFVSNGTHWFVTQRNFYEPQIITTDNT
FVSGNCDVVIGIVNNTVYDPLQPELDSFKEELDKYFKNHTSPDVDLGDISGINASVVNIQKEIDRLNEVA
KNLNESLIDLQELGKYEQYIKWPWYIWLGFIAGLIAIVMVTIMLCCMTSCCSCLKGCCSCGSCCKFDEDD
SEPVLKGVKLHYT

Figure S1. Primary structure of the spike protein (NCBI Reference Sequence: YP_009724390.1) of the Severe acute respiratory syndrome coronavirus 2 isolate Wuhan-Hu-1 (NCBI Reference Sequence: NC_045512). The RBD is shown in yellow and the B-epitopes computed by BepiPred in red.

1.1 Estimated Parameters

The model has been extensively used in the past so that many parameters have already been fixed (either by manual curation with literature information or by numerical estimation in generic settings - i.e., not pathogen-specific simulations). For the present study we calibrated only the parameters in table S1.

Table S1. Parameters estimated in the model

Name	Value	Meaning
p1	300	Vaccine dosage, i.e., adenoviral particles per microliter
p2	0.03	Probability proportional to the antigen persistence in antigen presenting cells
p3	148	Ratio of long lived plasma cells half life to half life of normal plasma cells
p4	0.695	Rate of release of spike proteins by adenoviral transfected cells i.e., muscle cells

1.2 Selecting the HLA haplotype

The computational model accounts for differences in the HLA haplotype when determining which peptides are presented by antigen-presenting cells. To this end, it takes in input a list of such peptides for each HLA molecule considered together with the propensity of each peptide to bind to it. This list is computed by using third-party immunoinformatics tools as described in the next section “Computing the peptide immunogenicity”. The “HLA haplotype freq search” in the “Allele Frequency Net Database” [2] was used in order to select two HLA-A, two HLA-B and two DRB alleles which are most prevalent in the caucasian phenotype [4]. The result pointed to the following alleles: HLA-A*02:01, HLA-A*24:02, HLA-B*35:01, HLA-B*40:02, DRB1*07:01 and DRB1*15:01.

1.3 Computing peptides immunogenicity

The strain of SARS-CoV-2 used in this study corresponds to the reference sequence NCBI Reference Sequence: NC_045512.2. The primary structure of the spike protein is the only molecule used to derive B epitopes and class I and II peptides. The spike protein (NCBI Reference Sequence: YP_009724390.1) is reported in Figure S1, with the RBD region evidenced in yellow and the B-epitopes computed with BepiPred (using the parameter EpitopeThreshold equal to 0.5, corresponding to specificity of 0.57 and sensitivity 0.58 [10]) shown in bold red. To identify cytotoxic T-cell peptides (CTL peptides) and helper T-cell peptides (HTL peptides) of the spike protein, we have employed two immunoinformatics tools:

- for the prediction of 9-mer long CTL peptides, the “ANN 4.0 prediction method” in the online tool MHC-I binding prediction of the IEDB Analysis Resource [20] was used to find peptides with affinity for the chosen set of HLA class I alleles (i.e., HLA-A*02:01, HLA-A*24:02, HLA-B*35:01 and HLA-B*40:02)[23, 19, 3]. The peptides were classified as strong, moderate and weak binders based on the peptide percentile rank and IC50 value. Peptides with IC50 values ¡50 nM were considered to have high affinity, ¡500 nM intermediate affinity and ¡5000 nM low affinity towards a particular HLA allele. Also, lower the percentile rank, greater is meant the affinity [23, 19, 3];
- for what concerns the HTL peptides, the NetMHCIIpan 3.2 server was used for the prediction of 9-mer long HTL peptides which had an affinity for the HLA class II alleles (i.e., DRB1*07:01 and DRB1*15:01) used in this study [14]. The predicted peptides were classified as strong, intermediate and non-binders based on the concept of percentile rank as given by NetMHCII with a threshold value set at 2, 10 and $> 10\%$, respectively. In other words, peptides with percentile rank ≤ 2 were considered as strong binders whereas a percentile rank between 2 and 10% designate moderate binders; peptides with percentile score > 10 are considered to be non-binders [14].

Since the list of CTL and HTL peptides and the relative affinity score is the same as in [7] we send the interested reader to the supplementary material of that work for further details.

1.4 Modeling adenoviral capsid immunogenicity

The anti-adenoviral antibody response was not simulated at the epitope level, but, in a simplification of a largely unknown scenario, the vaccine-induced antibody response to the adenoviral capsid was assumed to be proportional to the vaccine-induced antibody response to the Spike. To this end, the computational model included the rule that adenoviral vaccine-induced antibodies can bind to the adenoviral vaccine. When vaccine-induced antibodies bind to the vaccine and form immune complexes, there is a reduced transduction of muscle cells by the vaccine, and less Spike antigen is produced. To evaluate the effect of anti-adenoviral antibodies, in-silico immunization experiments were performed enabling or disabling the rule that adenoviral vaccine-induced antibodies can bind to the adenoviral vaccine. We found that the effect of the interval between doses is analogous in both experiments, as in both cases higher antibody responses are obtained at longer inter-dose intervals, as shown in Figure S2. With this in mind, in the present study, we choose to enable the rule that the vaccine induces anti-adenoviral antibodies, as this was demonstrated in a clinical trial [5] and is the more realistic scenario.

2 STATISTICAL METHODS

2.1 Approximate Bayesian Computation to estimate parameters

To calibrate the simulator with data from [11], we used the statistical method called Approximate Bayesian Computation (ABC, [9] [25] [24]) to estimate the following parameters of the computational model:

- θ_1 represents the persistence of phagocyted antigen before it is degraded in the cytosol of antigen presenting cells;
- θ_2 is the half life of plasma cells;
- θ_3 is the rate at which the Spike protein is produced in infected muscle cells.

Let us define
$x(0) = x_0$ the initial condition of the model;
v the vaccination schedule; we indicate with v_{1A} the vaccination schedule relative to the protocol 1A, v_{1B} for protocol 1B, and v_{1C} for protocol 1C;
$y(t) = M(\theta, x_0, v, t)$ denotes the trajectory of the model variables starting in the initial condition x_0 subjected to the vaccination schedule v. $M()$ is calculated running C-IMMSIM .

The ABC algorithm can then be formulated as follows:

Step 1: execute N times the simulation by sampling with the Latin Hypercube the space of meaningful parameters $\theta = (\theta_1, \theta_2, \theta_3)$;

Step 2: for each $i = 1, \ldots, N$ and for each $v \in \{v_{1A}, v_{1B}, v_{1C}\}$ compute J realizations of the stochastic model $y_j(t) = M_j(\theta_i, x_0, v, t)$ with $j = 1, \ldots, J$; scale $y_j(t)$ to be in $[0, 1]$;

Step 3: calculate $d_v = \left[\sum_t \left(\langle y(t)\rangle - y_t\right)^2\right]^{1/2}$ where $\langle y(t)\rangle$ is the average value, over the J simulations, of the trajectory point $y_j(t) = M_j(\theta_i, x_0, v, t)$ hence d_v is the residual

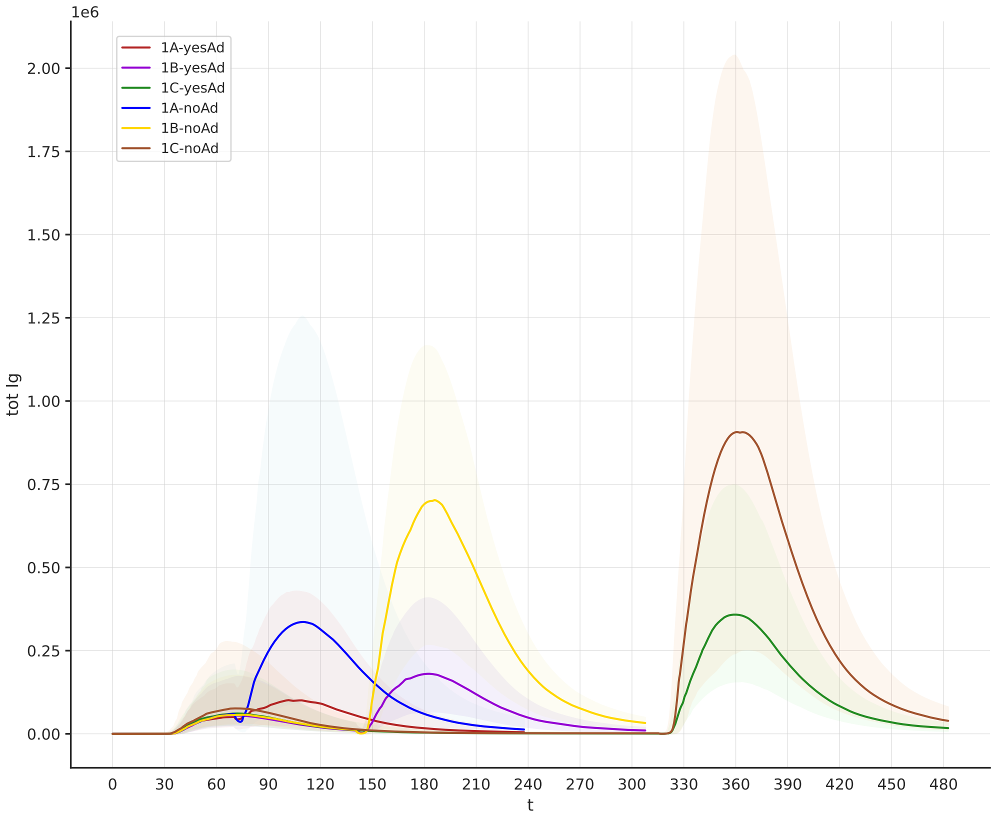

Figure S2. The graph reports the dynamic of IgG in two separate in-silico experiments, in which the antibodies induced by the vaccine were allowed (1A-yesAd, 1B-yesAd, 1C-yesAd) , or not allowed (1A-noAd, 1B-noAd, 1C-noAd) to bind the adenoviral capside. The time interval between the first and second dose was 10 weeks in 1A-yesAd and 1A-noAd, 20 weeks in 1B-yesAd and 1B-noAd, and 45 weeks in 1C-yesAd and 1C-noAd. The response to the second dose is lower in groups 1A-yesAd, 1B-yesAd and 1C-yesAd compared to 1A-noAd, 1B-noAd and 1C-noAD, indicating that vaccine-induced anti-adenoviral antibodies can reduce the immunogenicity of the second dose. On the other hand, the effect of the timing of the second dose on the magnitude of the response is similar in both experiments, with longer intervals resulting in higher responses in both situations.

sum of squares (RSS) computed summing all deviations from the available data points y_t;

Step 4: calculate $d_{1A} + d_{1B} + d_{1C}$ for each of the parameter considered θ and rank them from low to high, then pick the top 10% that minimise this sum. The distribution of these θs represents the posterior distribution of the parameters of interest.

2.2 Stepwise Regression

Stepwise regression is a statistical method to identify the most important variables among many of them when there is no theory that can help as a guide. The method is used to determine whether variables Ab(t1), Plb(t1), Th(t1), Tc(t1), and B(t1) can be used as explanatory variables for the increment of Ab induced by the second dose, that is $\Delta_{Ab} = Ab\,(t_m) - Ab\,(t_1)$.

There are three possible ways to implement it: the first consists of starting with a linear regression model containing only the constant term and, at each step, a regressor (or variable) is added to the model if it improves the fit evaluated using some criteria of fitness, such as the residual sum of squares; the second

algorithm consists of stating with a linear model which includes all the variables and at each step a variable is removed if its loss does not have a significant impact on the model fit; the third algorithm is a combination of the previous two. We have resorted to the third option for our analysis because it consists in the most complete one.

More details on these model selection algorithms can be found in [13].

2.3 Analyzing data by Principal Component Regression (PCR)

For the data analysis we have employed the Principal Component Regression (PCR), which is a regression model based on Principal Component Analysis (PCA) useful when dealing with multivariate data that exhibit multicollinearity. In particular, it consists of a linear regression where the dependent variable is explained/described as a linear combination of principal components. Principal components are orthogonal vectors that span the vector space generated by the original variables.
More formally, let $\boldsymbol{X} = (Ab(t_1), Plb(t_1), Th(t_1), Tc(t_1), B(t_1))$ be our regressors, $Y = Ab(t_m)$ be our dependent variable and $W = AX$ be the principal components, namely the i-th column of matrix A contains the loadings of the i-th component. Then, the principal components regression provides the following model

$$Y = \alpha_1 \boldsymbol{A}_1 \boldsymbol{X} + \alpha_2 \boldsymbol{A}_2 \boldsymbol{X} + \alpha_3 \boldsymbol{A}_3 \boldsymbol{X} + \alpha_4 \boldsymbol{A}_4 \boldsymbol{X} + \alpha_5 \boldsymbol{A}_5 \boldsymbol{X} \tag{S1}$$

where A_i refers to the i-th column of the matrix A (loadings of the i-th principal component) and α_i refers to the i-th regression coefficient. More details on PCR can be found in [13].

3 SUPPLEMENTARY FIGURES

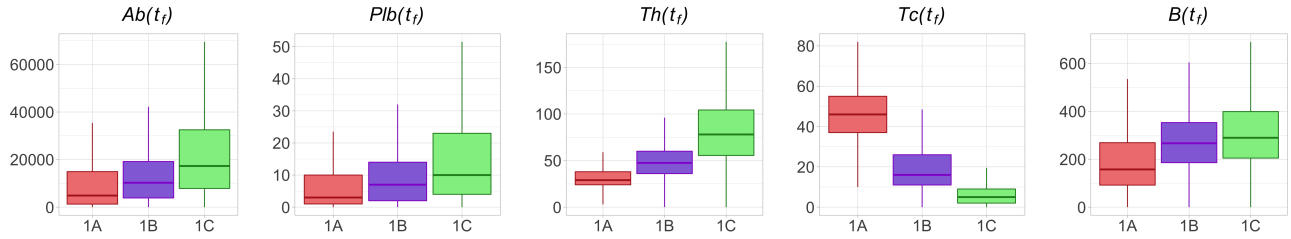

Figure S3. As the interval between the doses becomes longer, the humoral response (i.e., Ab, Pbl, B) and the T helper response (Th) to the second dose improve. The box plots show the median, IQR, and range of *Ab*, *Plb*, *Th*, *Tc* and *B* in treatment groups 1A, 1B and 1C at time t_f.

REFERENCES

[1]K. Abraham Peele, T. Srihansa, S. Krupanidhi, V. S. Ayyagari, and T. Venkateswarulu. Design of multi-epitope vaccine candidate against sars-cov-2: a in-silico study. *Journal of Biomolecular Structure and Dynamics*, 39(10):3793–3801, 2021.

[2]Allele Frequency Net Database. `http://www.allelefrequencies.net`, 2003–2020.

[3]M. Andreatta and M. Nielsen. Gapped sequence alignment using artificial neural networks: application to the mhc class i system. *Bioinformatics*, 32(4):511–517, 2016.

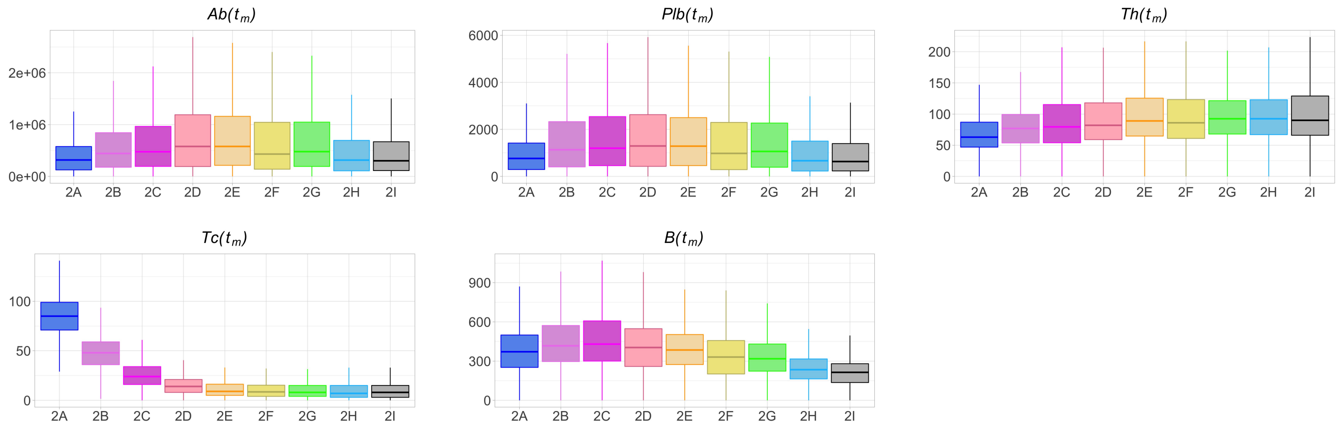

Figure S4. The optimal immunogenicity of the third dose is achieved over a large time window, spanning from 6 to 16 months after the second dose. The box plots show the median, IQR, and range of ab, plb, th,tc and b in treatment groups 2A-I at time t_m.

[4]M. S. Bardi, L. R. Jarduli, A. J. Jorge, R. B. O. G. Camargo, F. P. Carneiro, J. R. Gelinski, R. A. F. Silva, and E. L. Lavado. Hla-a, b and drb1 allele and haplotype frequencies in volunteer bone marrow donors from the north of parana state. *Revista brasileira de hematologia e hemoterapia*, 34:25–30, 2012.

[5]J. R. Barrett, S. Belij-Rammerstorfer, C. Dold, K. J. Ewer, P. M. Folegatti, C. Gilbride, R. Halkerston, J. Hill, D. Jenkin, L. Stockdale, et al. Phase 1/2 trial of sars-cov-2 vaccine chadox1 ncov-19 with a booster dose induces multifunctional antibody responses. *Nature medicine*, 27(2):279–288, 2021.

[6]F. Castiglione and F. Celada. *Immune system modelling and simulation*. CRC Press, 2015.

[7]F. Castiglione, D. Deb, A. P. Srivastava, P. Liò, and A. Liso. From infection to immunity: understanding the response to sars-cov2 through in-silico modeling. *Frontiers in immunology*, page 3433, 2021.

[8]F. Castiglione, D. Ghersi, and F. Celada. Computer modeling of clonal dominance: memory-anti-naive and its curbing by attrition. *Frontiers in immunology*, 10:1513, 2019.

[9]K. Csilléry, M. G. Blum, O. E. Gaggiotti, and O. François. Approximate bayesian computation (abc) in practice. *Trends in ecology & evolution*, 25(7):410–418, 2010.

[10]Bepipred-2.0: Prediction of potential linear b-cell epitopes. `http://web.archive.org/web/20080207010024/http://www.808multimedia.com/winnt/kernel.htm`. Accessed: 2022-15-07.

[11]A. Flaxman, N. G. Marchevsky, D. Jenkin, J. Aboagye, P. K. Aley, B. Angus, S. Belij-Rammerstorfer, S. Bibi, M. Bittaye, F. Cappuccini, et al. Reactogenicity and immunogenicity after a late second dose or a third dose of chadox1 ncov-19 in the uk: a substudy of two randomised controlled trials (cov001 and cov002). *The Lancet*, 398(10304):981–990, 2021.

[12]A. J. Greaney, A. N. Loes, K. H. Crawford, T. N. Starr, K. D. Malone, H. Y. Chu, and J. D. Bloom. Comprehensive mapping of mutations in the sars-cov-2 receptor-binding domain that affect recognition by polyclonal human plasma antibodies. *Cell host & microbe*, 29(3):463–476, 2021.

[13]T. Hastie, R. Tibshirani, J. H. Friedman, and J. H. Friedman. *The elements of statistical learning: data mining, inference, and prediction*, volume 2. Springer, 2009.

[14]K. K. Jensen, M. Andreatta, P. Marcatili, S. Buus, J. A. Greenbaum, Z. Yan, A. Sette, B. Peters, and M. Nielsen. Improved methods for predicting peptide binding affinity to mhc class ii molecules. *Immunology*, 154(3):394–406, 2018.

[15]M. C. Jespersen, B. Peters, M. Nielsen, and P. Marcatili. Bepipred-2.0: improving sequence-based b-cell epitope prediction using conformational epitopes. *Nucleic acids research*, 45(W1):W24–W29, 2017.

[16]T. Kar, U. Narsaria, S. Basak, D. Deb, F. Castiglione, D. M. Mueller, and A. P. Srivastava. A candidate multi-epitope vaccine against sars-cov-2. *Scientific reports*, 10(1):1–24, 2020.

[17]B. Kohler, R. Puzone, P. E. Seiden, and F. Celada. A systematic approach to vaccine complexity using an automaton model of the cellular and humoral immune system: I. viral characteristics and polarized responses. *Vaccine*, 19(7-8):862–876, 2000.

[18]O. Lund, M. Nielsen, C. Kesmir, A. G. Petersen, C. Lundegaard, P. Worning, C. Sylvester-Hvid, K. Lamberth, G. Røder, S. Justesen, et al. Definition of supertypes for hla molecules using clustering of specificity matrices. *Immunogenetics*, 55(12):797–810, 2004.

[19]C. Lundegaard, K. Lamberth, M. Harndahl, S. Buus, O. Lund, and M. Nielsen. Netmhc-3.0: accurate web accessible predictions of human, mouse and monkey mhc class i affinities for peptides of length 8–11. *Nucleic acids research*, 36(suppl_2):W509–W512, 2008.

[20]IEDB Analysis Resource. `http://tools.iedb.org/mhci/`, 2005–2022.

[21]M. Nielsen, C. Lundegaard, and O. Lund. Prediction of mhc class ii binding affinity using smm-align, a novel stabilization matrix alignment method. *BMC bioinformatics*, 8(1):1–12, 2007.

[22]M. Nielsen, C. Lundegaard, P. Worning, C. S. Hvid, K. Lamberth, S. Buus, S. Brunak, and O. Lund. Improved prediction of mhc class i and class ii epitopes using a novel gibbs sampling approach. *Bioinformatics*, 20(9):1388–1397, 2004.

[23]M. Nielsen, C. Lundegaard, P. Worning, S. L. Lauemøller, K. Lamberth, S. Buus, S. Brunak, and O. Lund. Reliable prediction of t-cell epitopes using neural networks with novel sequence representations. *Protein Science*, 12(5):1007–1017, 2003.

[24]M. Sunnåker, A. G. Busetto, E. Numminen, J. Corander, M. Foll, and C. Dessimoz. Approximate bayesian computation. *PLoS computational biology*, 9(1):e1002803, 2013.

[25]E. van der Vaart, M. A. Beaumont, A. S. Johnston, and R. M. Sibly. Calibration and evaluation of individual-based models using approximate bayesian computation. *Ecological Modelling*, 312:182–190, 2015.
